# Imaging Review of Procedural and Periprocedural Complications of Central Venous Lines, Percutaneous Intrathoracic Drains, and Nasogastric Tubes

**DOI:** 10.1155/2012/842138

**Published:** 2012-08-30

**Authors:** Hamdan Al-Jahdali, Klaus L. Irion, Carolyn Allen, Daniel Marafiga de Godoy, Ali Nawaz Khan

**Affiliations:** ^1^King Saud University for Health Sciences, King Adulaziz Medical City, Riyadh 11665, Saudi Arabia; ^2^The Cardiothoracic Centre Liverpool NHS Trust, The Royal Liverpool University Hospital, Liverpool L143LB, UK; ^3^North Manchester General Hospital, Pennine Acute NHS Trust, Manchester M85RB, UK; ^4^ULTRA-X, 97.060.380 Sao Jose do Rio Preto, SP, Brazil

## Abstract

Placements of central venous lines (CVC), percutaneous intrathoracic drains (ITDs), and nasogastric tubes (NGTs) are some of the most common interventional procedures performed on patients that are unconscious and in almost all intensive care/high dependency patients in one form or the other. These are standard procedures within the remit of physicians, and other trained health professionals. Procedural complications may occur in 7%–15% of patients depending upon the intervention and experience of the operator. Most complications are minor, but other serious complications may add significantly to morbidity and even mortality of already compromised patients. Imaging findings are the key to the detection of misplaced lines, and tubes and their prompt recognition are vital to avoid harm to the patient. It is, therefore, pertinent that healthcare professionals who perform these procedures are familiar with imaging complications of these procedures. Here, we present the imaging characteristics of procedural complications.

## 1. Introduction

Placements of CVC, ITD, and NGT are the most common interventional procedures performed on the seriously ill postoperative patients that are unconscious and in almost all intensive care/high dependency patients in one form or the other. These are standard procedures within the remit of physicians, and other trained health professionals. Procedural complications are significant, but most are classed as minor. However, complications that are more serious occur. Avoiding complications from CVC, ITD, and NGT placement is a subject of particular concern and interest in the ongoing era of quality and safety culture. Expected benefits need to outweigh the risk of complications. The corner stone in the detection of these complications is readily available appropriate imaging during and after the procedures.

Portable chest radiography is an essential clinical component of patient care in the intensive care unit. Routine use not only shows unexpected cardiopulmonary abnormalities, but also plays an important role in the detection of malposition of various lines and tubes allowing prompt action [1, 2]. Occasionally computed tomography (CT) is indicated. Until recently, the main stay of detecting complications was a standard chest (CXR) and/or an abdominal X-ray (AXR). However, the growing importance of bedside ultrasound as a tool of superior accuracy in diagnosing periprocedural complications needs to be emphasized [3, 4]. Sonography is taking a greater share in the management of CVCs, NGTs, and percutaneous thoracic intervention resulting in a diminishing role for a CXR. Ultrasound guidance is an accurate method of CVC misplacement detection leading some authors to opt for total abolishment of routine CXR after CVC placement [5]. Similarly, ultrasonography as an alternative method of confirming proper nasogastric tube placement has been proposed [6]. It has been suggested that the remaining indication for a CXR seems to be detection of the catheter tip malposition [7–9]. Studies from Italy [10] and France [11] suggest that ultrasound is an accurate method of CVC misplacement detection, leading some authors to opt for total abolishment of routine CXR after CVC placement.

The physician or other healthcare professionals caring for these critically ill patients should be familiar with imaging features of complications caused to take the immediate and appropriate action. Here, we present imaging findings of minor and life threatening major complications.

## 2. Procedures Complications

Procedures complications from intrathoracic CVC, ITD, and NGT placement are significant, but most are considered minor. However, complications that are more serious occur. Avoiding complications from CVC, ITD, and NGT placement is a subject of concern and interest in the ongoing era of quality and safety culture. Expected benefits need to outweigh the risk of complications. The corner stone in the detection of these complications is readily available appropriate imaging during and after the procedures.

The physician or other healthcare professionals caring for these critically ill patients should be familiar with imaging features of complications caused to take the immediate and appropriate action. The Do's and Do not's of CVC, NGT, and ITB placements are summarised in Tables 1, 2, and 3 and which, form the basis of avoiding complications. Here, we present imaging findings of minor and life threatening major complications.

Image-guided placements reduce the risk of complications. Sonography is taking a greater share in the management of CVCs, NG tubes, and percutaneous thoracic intervention resulting in a diminishing role for a CXR. Ultrasound is an accurate method of CVC misplacement detection, leading some authors to opt for total abolishment of routine CXR after CVC placement [5]. Similarly, ultrasonography as an alternative method of confirming proper nasogastric tube placement has been proposed [6].

### 2.1. Central Venous Lines (Catheter) (CVC)

CVC is placed in one of the main veins including the jugular (internal or external), the subclavian, or the femoral vein. CVC lines are used to administer medications or fluids, obtain blood samples specifically for “mixed venous oxygen saturation,” and directly obtain cardiovascular measurements such as the central venous pressure. There are several types of central venous catheters in use for a variety of indications. The meta-analysis study and National Institute of Clinical Excellence recommend that CVC lines be ultrasound guided [12, 13].

Central venous line insertion may cause a number of complications. The benefit needs to outweigh the risk of those complications. Complications related to placement occur in approximately 15% [14–16]. Procedural complications occur in 5 to 19% of patients [5, 6, 15, 16]. Infectious complications occur in 5 to 26% [5, 7, 15, 17], whilst thrombotic complications occur in 2 to 26% [5, 15]. The do's and do not's of CVP lines are summarized in Table 1.

#### 2.1.1. Incorrectly Placed Catheters

**Figure 1:**
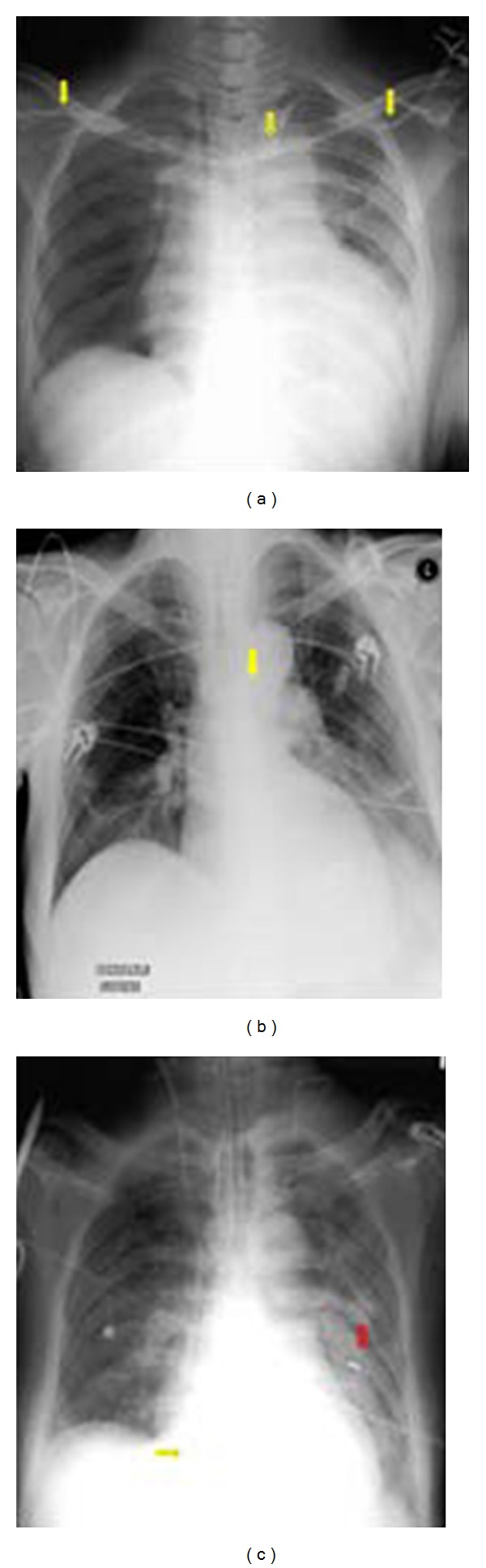
Portable AP radiographs on three different patients showing malpositioned central venous lines. (a) It shows that the IV line has crossed from the left axillary to the right axillary vein (arrows). (b) It shows the central line placed via the right jugular vein has entered the left brachiocephalic vein (arrow). (c) It shows that the tip of the central line has entered the inferior vena cava (yellow arrow), whilst the NGT has entered the left lower lobe bronchus associated with a left basal pneumonia.

**Figure 2:**
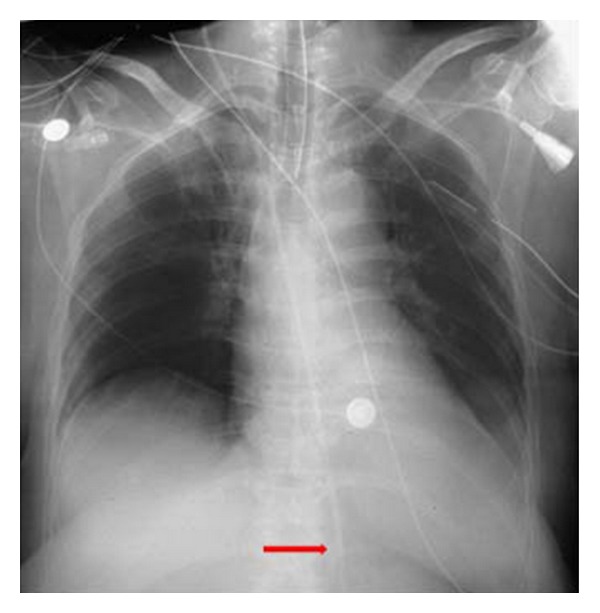
A CXR shows that an intravenous line placed via the right internal jugular vein as entered the azygos venous system.

**Figure 3:**
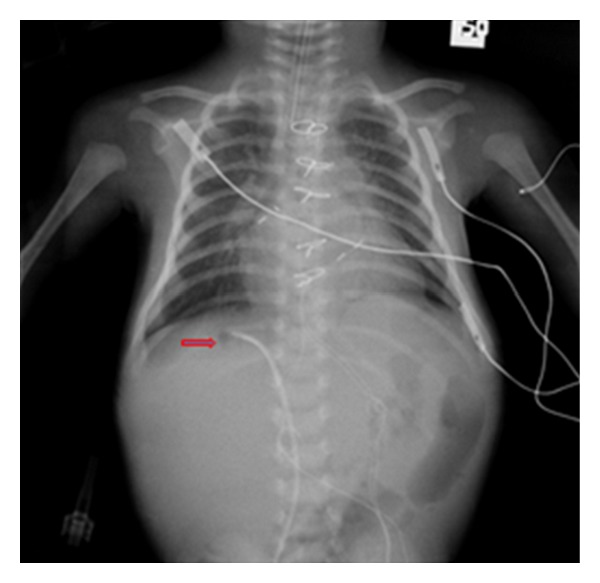
A chest radiograph on 2-month-old following cardiac surgery. A difficult venous access prompted a right femoral vein access. The tip of the catheter has entered the right hepatic vein. Note the air at the tip of the catheter, which is a potential for air embolism (arrow).

**Figure 4:**
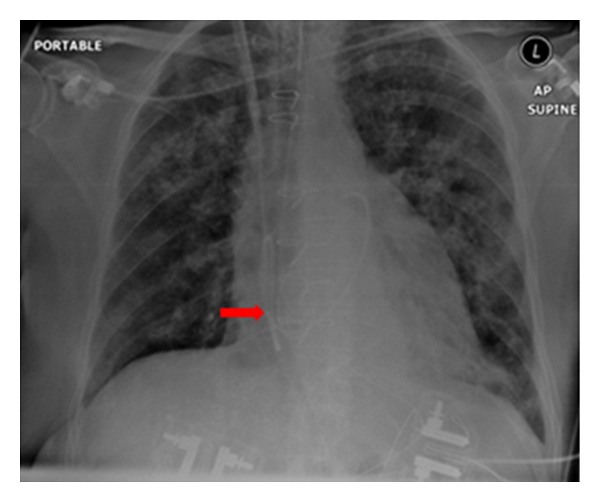
A CXR showing a Swan Ganz catheter forming a loop in the IVC (arrow).

**Figure 5:**
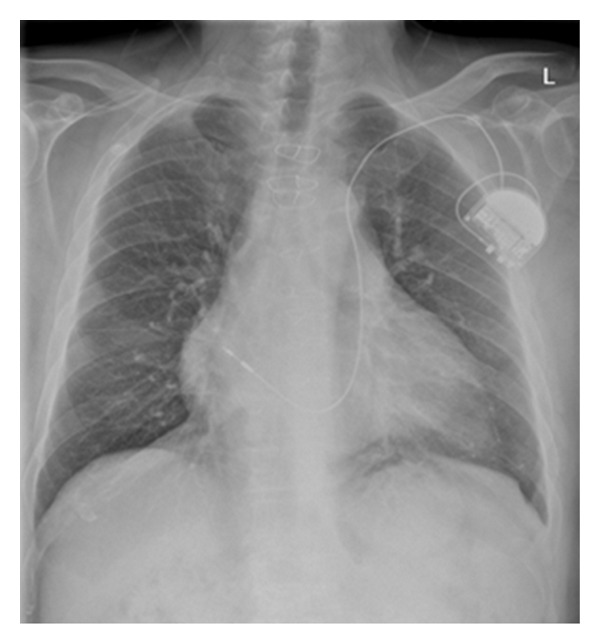
Intravenous lines can appear in unusual locations. This patient had a pacemaker wire placed via the left subclavian vein. The wire follows an unusual course via a left-sided superior vena cava. Anatomical vascular variants should be considered when ever an IV line follows an unfamiliar path.

Accurately catheter tip placement of the intravenous catheter is crucial to its safe function. The distal points of short-term catheters are usually placed within the SVC, and long-term catheters are placed in the superior vena cava or just within the right atrium. Malposition as detected on chest X-rays in 3–14% of patients, with the catheter tip located in the internal jugular vein, opposite subclavian vein, axillary vein, internal thoracic vein, azygos vein, hemiazygos vein, or pericardiophrenic vein [18–20] (Figures 1, 2, 3, 4, and 5). Malposition in these locations is associated with higher complication rates such as pain, malfunction, phlebitis, and thrombosis [21, 22]. Such malpositioned catheters are associated with a high prevalence of pain, thrombosis, phlebitis, and malfunctions. Mention needs to be made of a persistent left superior vena cava [23]. A catheter would show in an abnormal position when placed in a persistent left superior vena cava when assessed on a standard A-P chest X-ray (Figure 5). A quick squirt of contrast through the line would confirm the location of the catheter.

**Figure 6:**
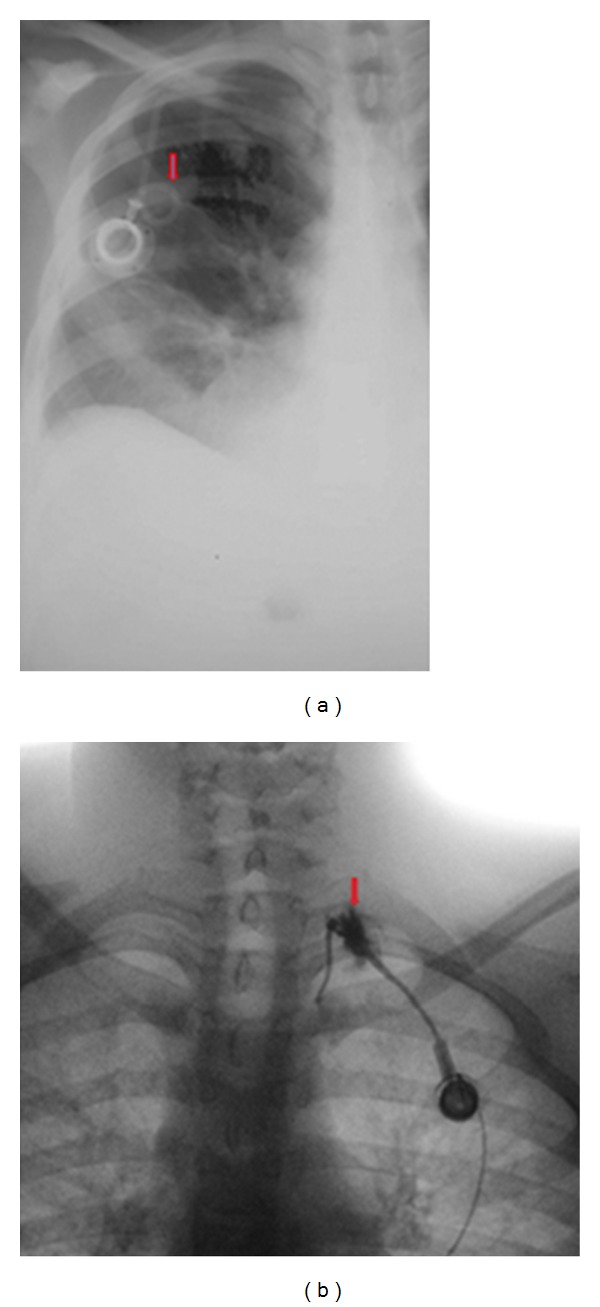
Two patients with surgically installed subcutaneous ports anterior thorax before chemotherapy starts. The line will allow treatments to be given and blood samples obtained without being “stuck” with a needle. At the end of the treatment, the central line is removed. (a) It shows a looped catheter and (b) shows dissection of the vessel.

**Figure 7:**
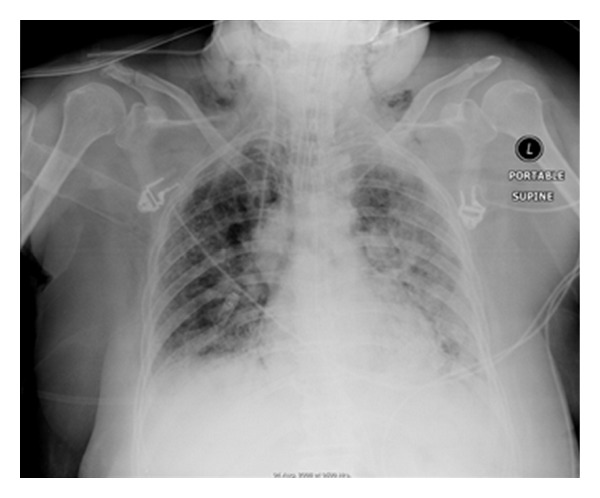
A CXR showing a pneumothorax following insertion of an intravenous line via the right jugular vein. Note the surgical emphysema in the neck.

Extravascular location of catheter tips can cause extravasation, pneumothorax, hemothorax, or hemomediastinum (Figures 6 and 7). A CXR may confirm the position of the catheter however; sometimes a lateral radiograph may be required for proper location. Imaging is necessary following every placement of an intravenous line as entry of solutions into the mediastinum or pleural space may have disastrous consequences. Failure to obtain free reflux of blood from the catheter at the time of insertion and following insertion should raise the possibility of abnormal location of the catheter tip.

When considering ultrasound-guided vascular puncture techniques many alternatives are available, which include low or posterior approaches of the internal jugular vein, puncture of the subclavian vein and its variant in children, infraclavicular access to the axillary vein, and also more peripheral punctures of the basilic, brachial, and cephalic veins [24].

Complications associated with insertion of central lines include bleeding, pneumothorax, hemothorax, arterial cannulation, and catheter displacement or break. Catheter breakage occurs mainly with extensive catheter manipulation during difficult insertion. Another cause of catheter breakage according to the literature is the so-called “pinch-off” syndrome, where the CVC is compressed between the first rib and the clavicle, a phenomenon occurring in approximately 1% of all long-term CVCs'. This complication may result in catheter breakage with subsequent embolization of the distal portion [25].

Catheter kinking may also hinder flow, and it occurs most often in obese patients when the skin is drawn laterally and inferiorly during needle insertion. Following skin release, a right angle kink of the catheter can occur as it exits from under the clavicle running through the subcutaneous tissue to the exit site with another right angle and again at the skin, forming a Z-shaped deformity [13, 26] (Figure 6).

#### 2.1.2. Arterial Puncture

Internal jugular vein catheterization is associated with a high rate of successful catheter placement. However, serious complications arise with internal jugular catheterization such as carotid artery puncture, pneumothorax, vessel erosion, thrombosis, and airway obstruction and infection. Rare complications include thyrocervical trunk, pseudoaneurysm, and fistula [27].

Inadvertent arterial puncture occurs in approximately of 3%–10%, usually does not result in serious consequences, but can occasionally lead to devastating complications (e.g., hematoma, pseudoaneurysm, or stroke) particularly if it goes unrecognized and a large-bore dilator or catheter is inserted [28]. The risk of CVC insertion-associated severe bleeding even in coagulopathic or thrombocytopenic patients is low (<5%) [29].

#### 2.1.3. Pneumothorax/Hemothorax

Pneumothorax is a common complication of subclavian vein catheterization and range from 0.1 to 15% [28, 30, 31] (Figure 7). Thin, malnourished patients, and patients with COPD, and those on high-pressure ventilatory support are at an increased risk. The pneumothorax is usually small, and most patients remain asymptomatic and resolve without intervention. Occasionally, a pneumothorax is not seen on the first post-insertion CXR and becomes a few days later. Difficult insertions, particularly when several passes are made and when air is aspirated and in patients that complain of pleuritic pain a normal initial CXR may require a repeat CXR in 6–12 hours post-insertion. Most delayed pneumothoraces resolve spontaneously. 

Spiliotis and associates conducted a prospective study of 343 catheter placements via the subclavian vein describe a 2.2% delayed pneumothorax rate recognised 48 and 72 hours after the catheter placement [26, 32].

In another retrospective study, one hundred fourteen patients having 121 subclavian venipunctures were studied, where eight pneumothoraces occurred (6.6%) and were most frequent after the insertion of large catheters, or when the subclavian area was distorted by previous venipuncture or radiation. Delayed pneumothorax occurred in five patients detected 8–96 hours following the venipuncture. All patients in the study with delayed pneumothoraces required a tube thoracotomy [27, 33].

#### 2.1.4. Chylothorax

A chylothorax due thoracic duct injury may follow a subclavian vein or jugular vein catheterization is uncommon complication and reported in 1–4.2% [34]. This complication is rarely recognised immediately, but leakage of lymphatic fluid at the puncture site may become apparent over time, making dressing care difficult, or a CXR may reveal nonspecific pleural effusion. Rarely a fluctuant swelling is seen at the root of the neck. 

#### 2.1.5. Air Embolism

**Figure 8:**
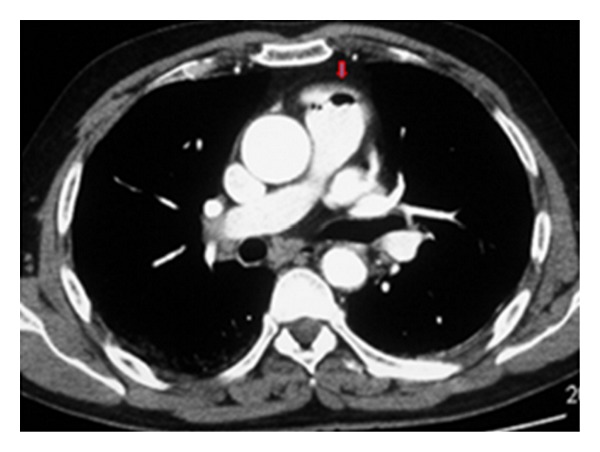
An axial CT scan shows air embolism with air in the pulmonary artery following the withdrawal of a central venous line (arrow).

Air embolism is uncommon however, potentially fatal complications of an intravenous line are possible [35]. Fatalities have been reported following intravenous administration of 100–200 mL of air. Several cases of death following air embolism have been reported associated with central venous lines. Air embolism may occur during venipuncture, during the changing of intravenous tubing, and accidental disconnection of intravenous tubing from the central venous catheter. Symptoms are dependent on the amount and rate at which air is aspirated. Patients may suffer from dyspnea, chest pain, and cyanosis. The patient may become disoriented and comatose. Physical examination may reveal tachycardia and hypotension. The diagnosis can be confirmed by a cross-table CXR with the right side up. Air may be seen in the pulmonary arteries and/or the right ventricle [35–38] (Figure 8).

### 2.2. Percutaneous Thoracic Drains

There is a variety of indications for placement of chest drains. Physicians and technologists from many specialties are trained on the methods that can allow them to safely perform tube thoracostomy with 3% early and 8% late complications.

The National Patient Safety Agency (UK) reported 2152 complications that were related to chest drain placements between January 2005 and March 2008. Fifteen serious complications and 12 fatalities were reported. Most serious complications were related to the site of drain insertion. This issue has raised concern regarding the risk of incorrect placement of chest drains and adequacy of training amongst those responsible for chest drain insertion [34–37, 39–43].

The use of a trocar has been linked to a significant incidence of intraparenchymal and intrafissural insertion of chest drains. Remérand and his colleagues in a review of 122 patients with chest drain insertion followed by CT found 21% of drains to be intrafissural and 9% to be intraparenchymal with the only predicting factor associated with the risk of malposition was the use of a trocar for the insertion of the chest tube [43].

British Thoracic Society (BTS) guidelines recommend that placement of a chest drain is indicated for a malignant pleural effusion, empyema, traumatic hemothorax, some pneumothoraces, and in some postoperative states, such as after cardiac surgery. BTS guidelines recommend that all elective drains should be inserted in the “triangle of safety”. The triangle of safety is defined as the area bordered by the anterior margin of latissimus dorsi, the lateral edge of pectoralis major, and a line superior to the horizontal level of the nipple with the apex below the axilla. Inappropriate insertion of a chest drain can cause serious harm or even death. Although placement of a chest drain in the “safe triangle” is regarded, standard, other location may be considered. Placement of a drain in the midclavicular line in the second intercostal space may provide an alternative insertion site in the presence of an apical pneumothorax. This route is not routinely taken as it may be uncomfortable for the patient and may produce an unsightly scar [43–45].

Alternative insertion sites are also considered as in loculated pleural effusions where choosing a posterior site may be more appropriate. When draining loculated pleural effusions ultrasound guidance may provide a safer approach. Image-guided thoracocentesis has a higher success rate and lower complication rate [46–50].

Placement of chest drains is a common hospital procedure; most are placed safely without complications. Nevertheless serious and fatal complications may occur. All manner of misplaced chest drains has been described. Perforation of the pericardium, cardiac chambers, injury to the aorta, thoracic duct and stomach, spleen, and liver has been described. A diaphragmatic rupture has been misinterpreted as a pneumothorax, and stomach contents have been drained [45, 46] (Figures 9, 10, 11, 12, 13, 14, 15, 16, 17, 18, 19, 20, 21, 22, 23, 24, 25, 26, 27, 28, 29 and 30). It is important that a pseudopneumothorax and lung bullae are recognized to prevent inadvertent placement chest drain placement. The do's and do not's of percutaneous chest drains are summarized in Table 2 [44, 50].

### 2.3. The Nasogastric Tube

Insertion of an NGT is a common clinical procedure. The NGT provides access to stomach for several diagnostic and therapeutic indications. The diagnostic applications include evaluation of upper gastrointestinal bleed, aspiration of gastric contents, and administration of radiographic contrast to the GI tract. Therapeutic applications include gastric decompression, aspiration of gastric contents of recently ingested toxins, administration of medication, and feeding and bowel irrigation. NGT insertion can be uncomfortable with inadequate local anesthetic to the nasal passages and lack of instruction to patients on how to cooperate with the operator during the procedure. Inexperienced or inaccurate placement may not only be distressing to patients, but also could cause significant harm or even death.

The clinical signs of NGT misplacement in intensive care patients may be absent or misleading. Chest radiography is accurate in the detection of misplaced NGT. Chest radiography is particularly helpful in detecting NGT misplacement. The complication rates associated with fine bore NGTs varies from 0.35 to 8% [45, 51] (Figures 31, 32, 33, 34, 35, 36, 37, 38, 39, 40, and 41). The Do's and Do not's of NGT are summarized in Table 3.

#### 2.3.1. Respiratory

**Figure 34:**
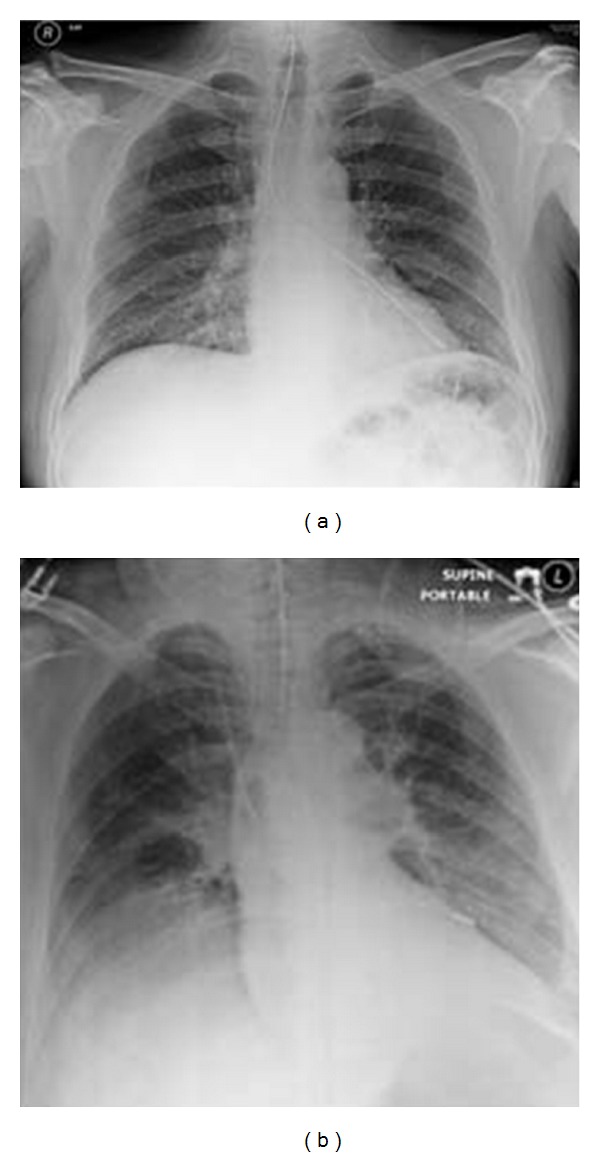
The NGT has entered the left main lower lobe bronchus in two different patients. (b) shows aspiration pneumonia at both lung bases more pronounced on the left.

**Figure 35:**
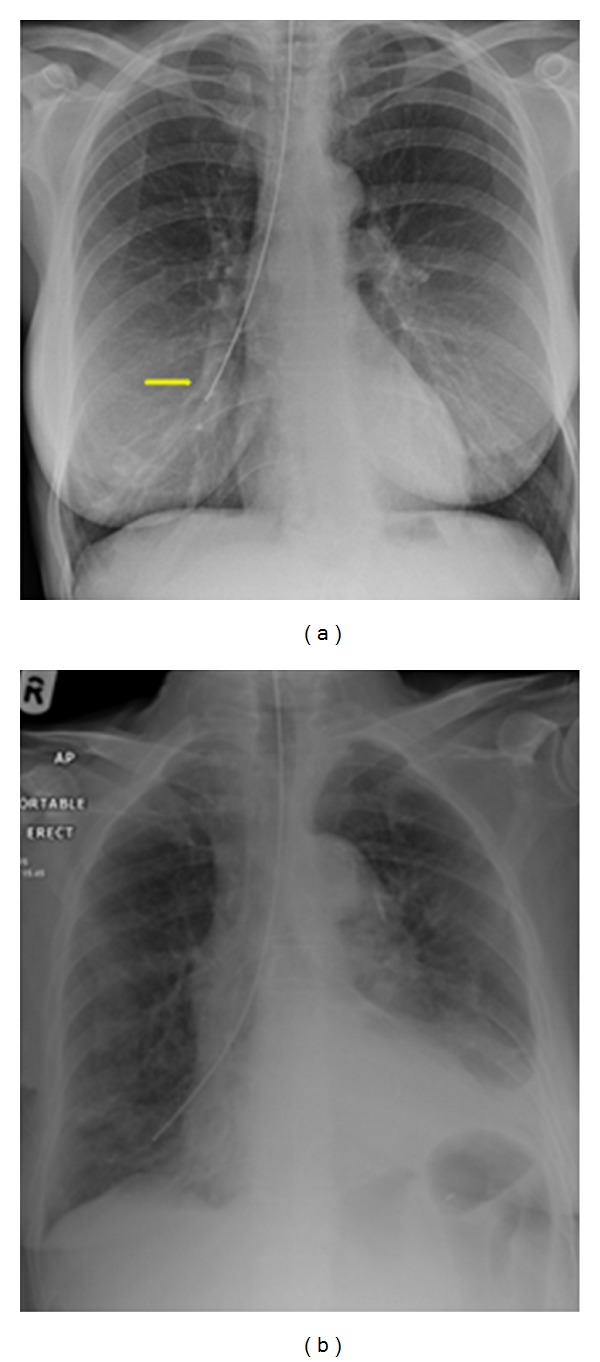
The NGT has entered the main right lobe bronchus in two patients. The patient in (b) already had left basal pneumonia; delivery of fluids down the right lower lobe bronchus would have resulted in disastrous consequences of the position of the NGT that had gone unrecognized.

**Figure 36:**
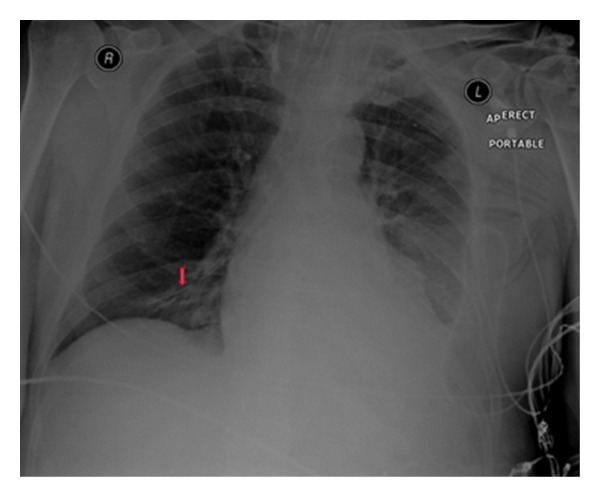
The quality of a portable AP radiograph is not always optimal because of the technical factors prevailing. The tip of the NGT in the lower lobe bronchus is obscured by lung markings (arrow).

**Figure 37:**
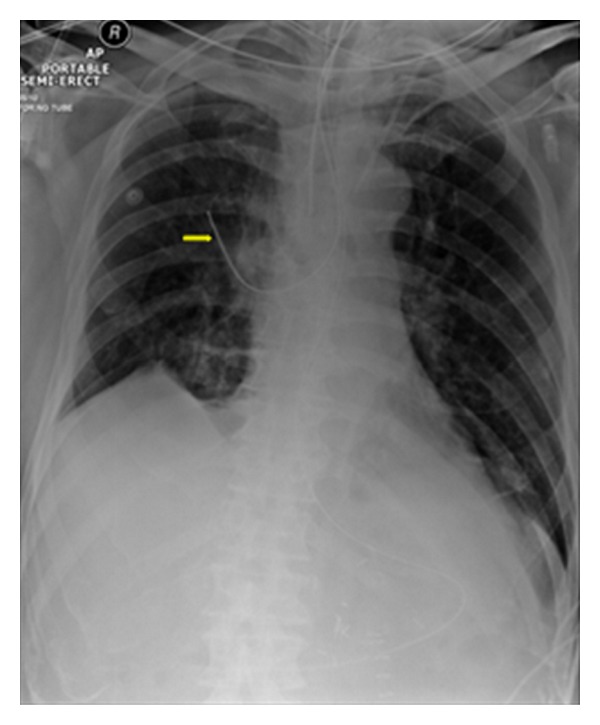
The tip of the NGT has entered the right upper lobe bronchus.

**Figure 38:**
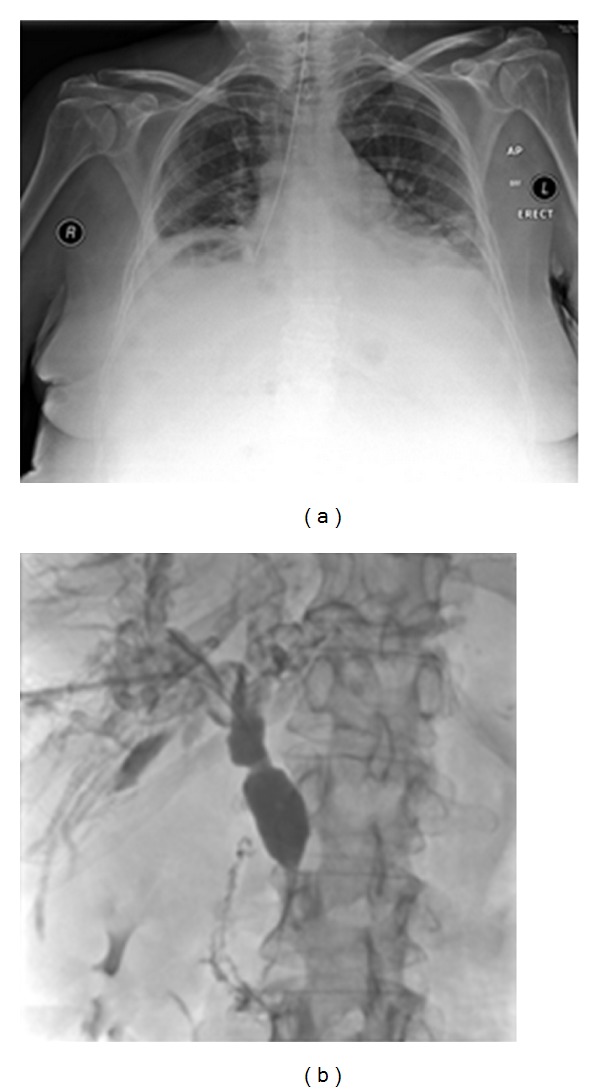
A CXR shows entry of the NGT into the right lower lobe bronchus. The patient was severely ill and went into respiratory distress following delivery of fluids down the NGT. There was a delay in the diagnosis of the patient developing an abscess at the right lung base. The patient had a cholangiocarcinoma and had an unsuccessful attempt at external biliary drainage (PTC (a)).

**Figure 39:**
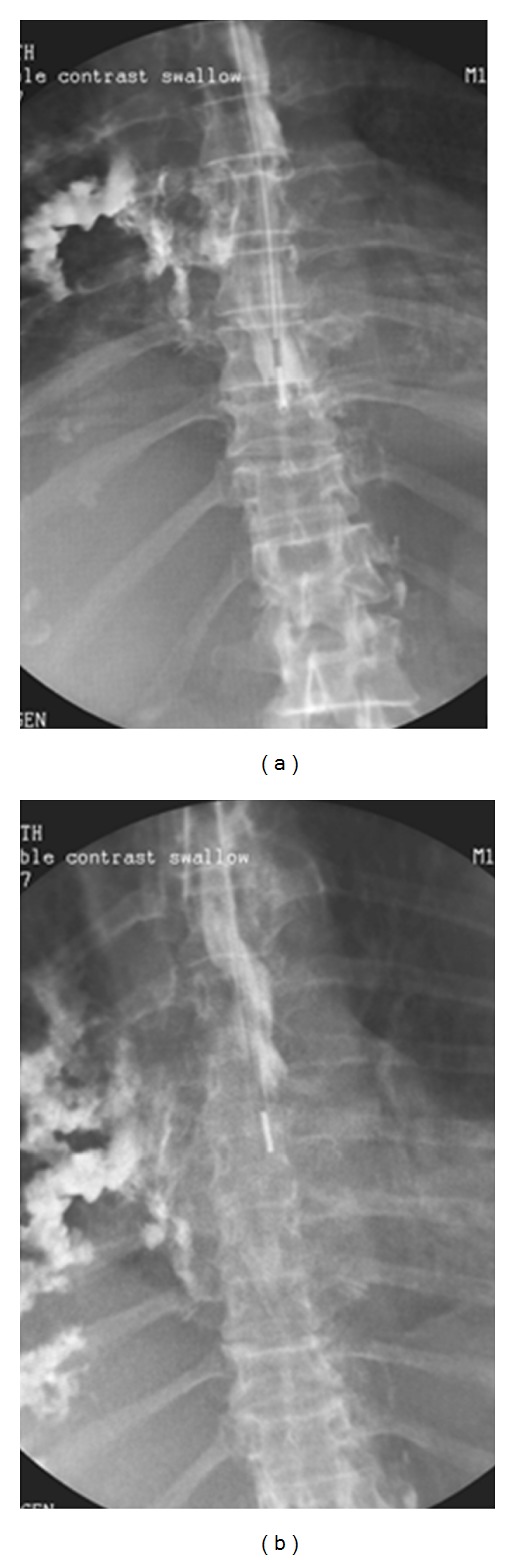
Two images from a water-soluble contrast swallow showing an esophageal perforation from an NGT in this case with esophageal varices.

Most complications related to NGTs are due to inadvertent placement in the respiratory tract (Figures 34–39). A tube with feeding ports in the esophagus significantly increases the risk for aspiration, as does the displacement of a small bowel tube into the stomach of a patient with significantly slowed gastric motility.

Rassias reported a 2% incidence of tracheopulmonary complications among 740 tube insertions and 0.3% died from the complications [47, 52].

Complications related to malpositioned feeding tubes are usually preventable. Some pulmonary complications related to NG misplacements can be particularly significant. Bronchial placement leads to atelectasis, pneumonia, and eventually developing into lung abscess. Other respiratory complications include bronchial perforation, perforation into the pleural cavity and pneumothorax, hydrothorax, rarely pulmonary hemorrhage, and pleural knotting of the NGT [51–55].

### 2.4. Intravascular Complications

Accidental intravascular placement in right internal jugular vein down into the right atrium has been reported. Placement of an NG tube into an aberrant right subclavian artery through an esophageal erosion has also occurred [56, 57]. Feeding tubes should be avoided in those known to have an aberrant right subclavian artery. Fatal hematemesis has been reported [56, 57].

#### 2.4.1. Enteral Complication

NGT knotting is a rare complication, can occur in various locations, and case reports describe knotting/impaction in the nasopharynx and beyond the pylorus. Other NG tube complications include re-entry of the esophagus following a loop in the stomach, tube breakage, tube blockage, and rupture with syringing and gastrointestinal perforation. Esophageal perforation and subsequent mediastinitis may occur [58, 59].

#### 2.4.2. Intracranial Entry

Intracranial placement of an NG tube has occurred following repair of choanal atresia, transsphenoidal surgery and maxillofacial trauma [60, 61].

## 3. Summary

Procedural complications may occur in 7%–15% of patients during placement of CVC, ITD, and NGT. Serious complications may add significantly to morbidity and mortality of already compromised patients. Imaging findings are the key to the identification of misplaced lines, and tubes, and their prompt recognition are essential to avoid harm to the patient. All those that indulge performing the procedures to avoid harm to patients must recognize these imaging findings. Imaging characteristics of these complications are described, and ways to identify them are discussed.

## Figures and Tables

**Figure 9:**
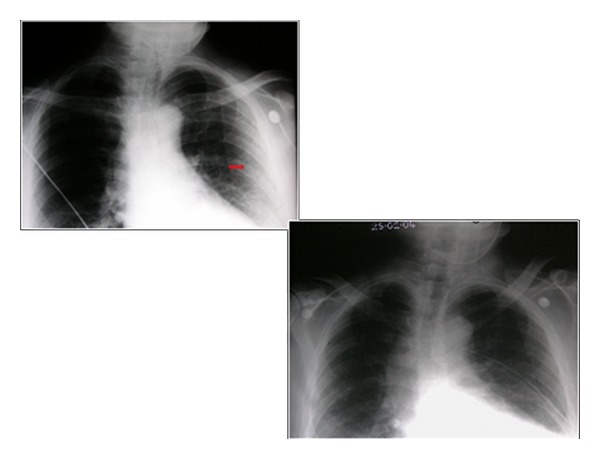
A CXR showing a chest drain placed into a left-sided pseudopneumothorax. Before placement of chest drains, it is essential that pneumothorax mimics be recognized.

**Figure 10:**
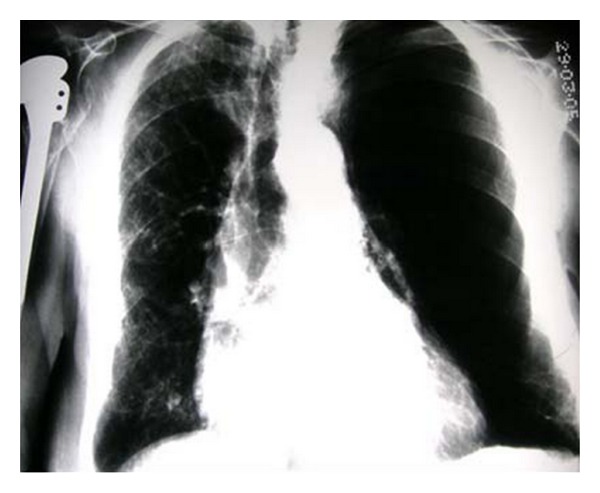
A CXR on a patient with COPD that had undergone a recent shoulder arthroplasty presented with acute shortness of breath. Diagnosis of left-sided pneumothorax was made clinically and from the CXR. A question of bullous emphysema was raised so an urgent CT scan was arranged. See Figure 12.

**Figure 11:**
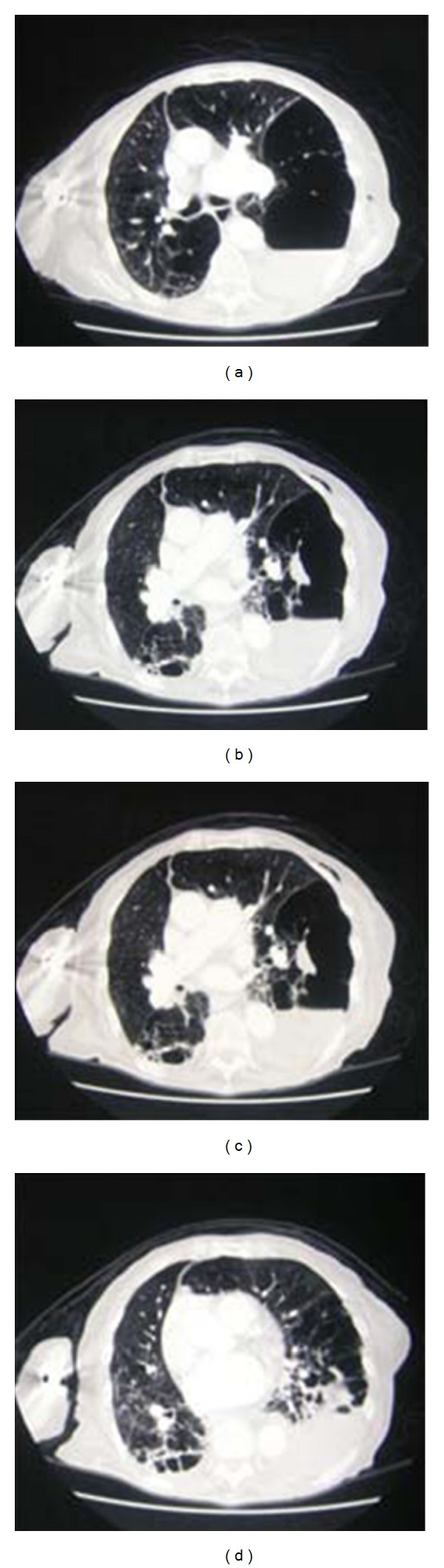
A CT scan on the same patient as in Figure 11 shows extensive bullous emphysema. The punctured bulla contains an air/fluid.

**Figure 12:**
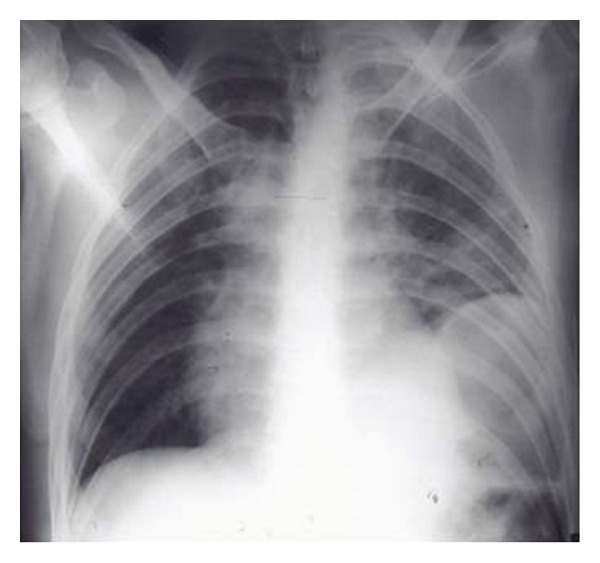
A CXR on a patient with thoracic trauma following a road traffic accident. Diagnosis of a hemopneumothorax was made, but before proceeding to a chest drain placement, a CT scan was obtained as a part of a work up for multiple traumas, which showed a diaphragmatic rupture and herniation of stomach into the left hemithorax explaining the air/fluid at the left lung base.

**Figure 13:**
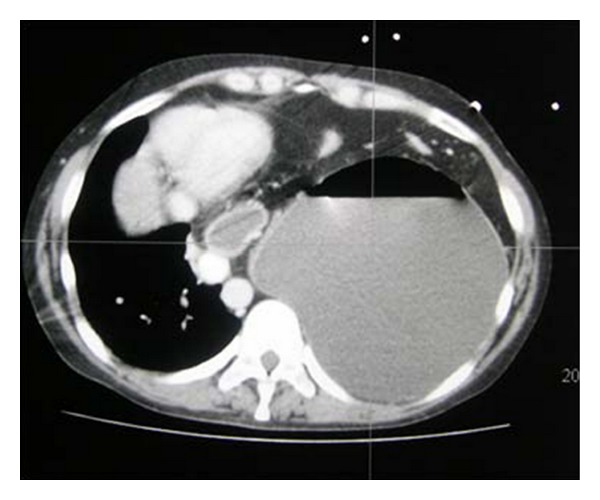
Axial CT on the same patient as Figure 13 showing diaphragmatic rupture and herniation of the stomach into the left hemithorax.

**Figure 14:**
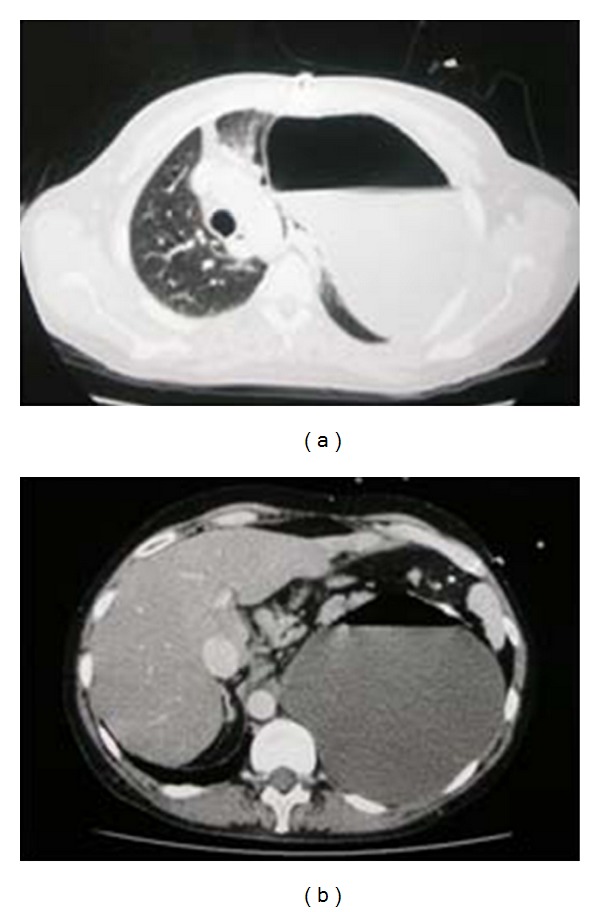
Axial CT scans on the same patient as in Figures 13 and 14 showing diaphragmatic rupture and herniation of the stomach into the left hemithorax.

**Figure 15:**
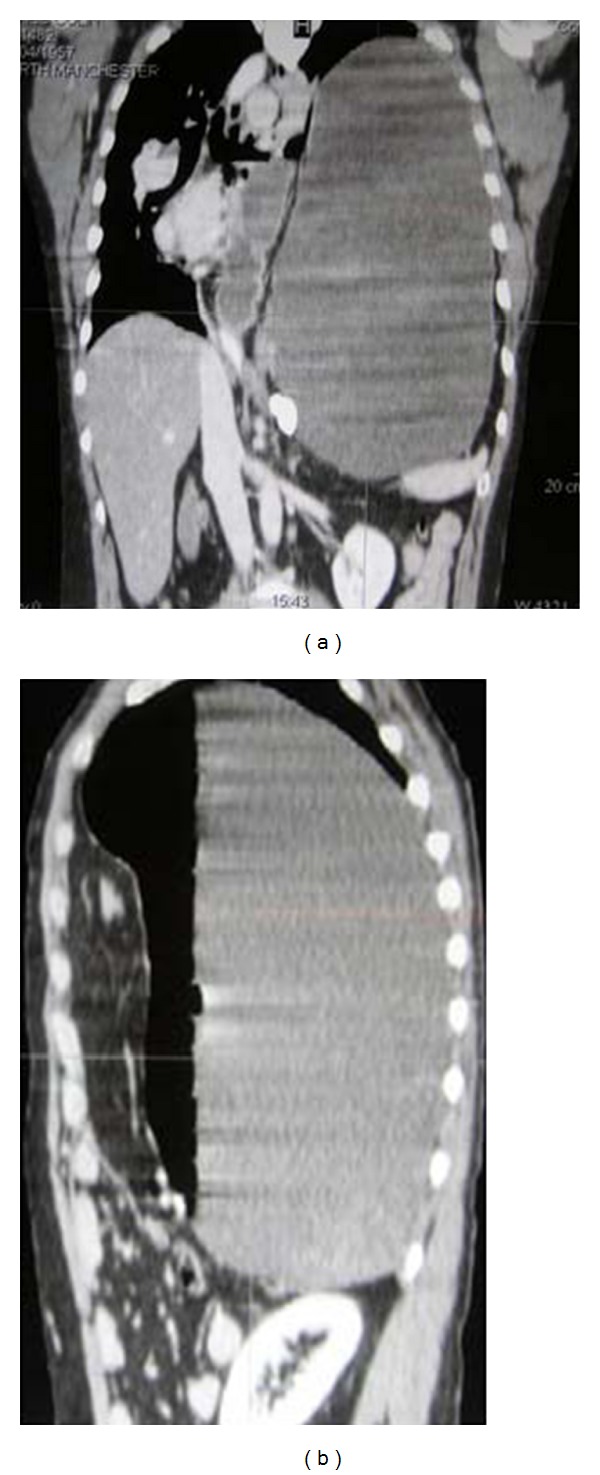
Coronal and sagittal CT reconstruction on the same patient as Figures 13, 14, and 15 showing diaphragmatic rupture and herniation of the stomach into the left hemithorax.

**Figure 16:**
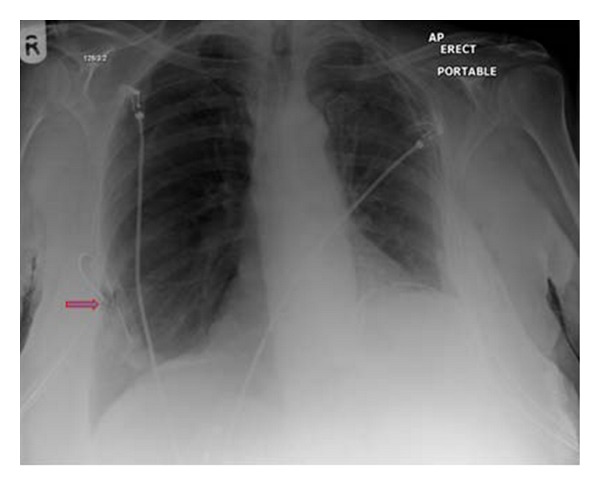
A kink in a chest drain that failed to evacuate the right-sided pleural effusion.

**Figure 17:**
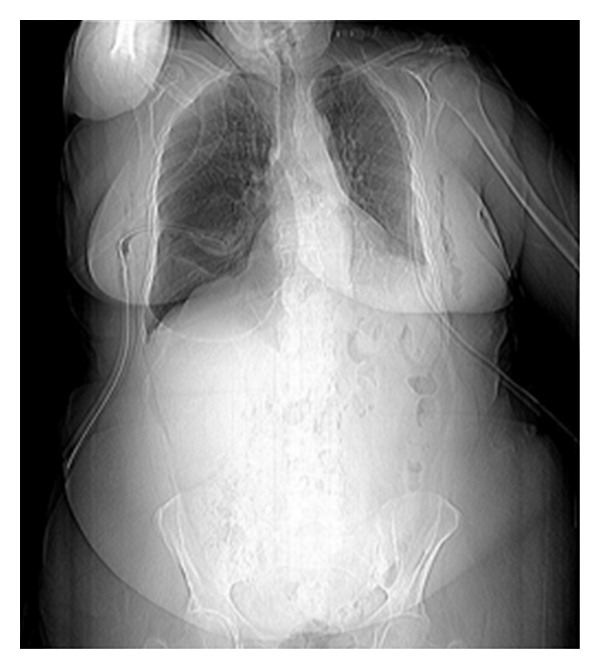
A CT Scanogram showing bilateral chest drains the right drain is inappropriately placed in a right basal bulla.

**Figure 18:**
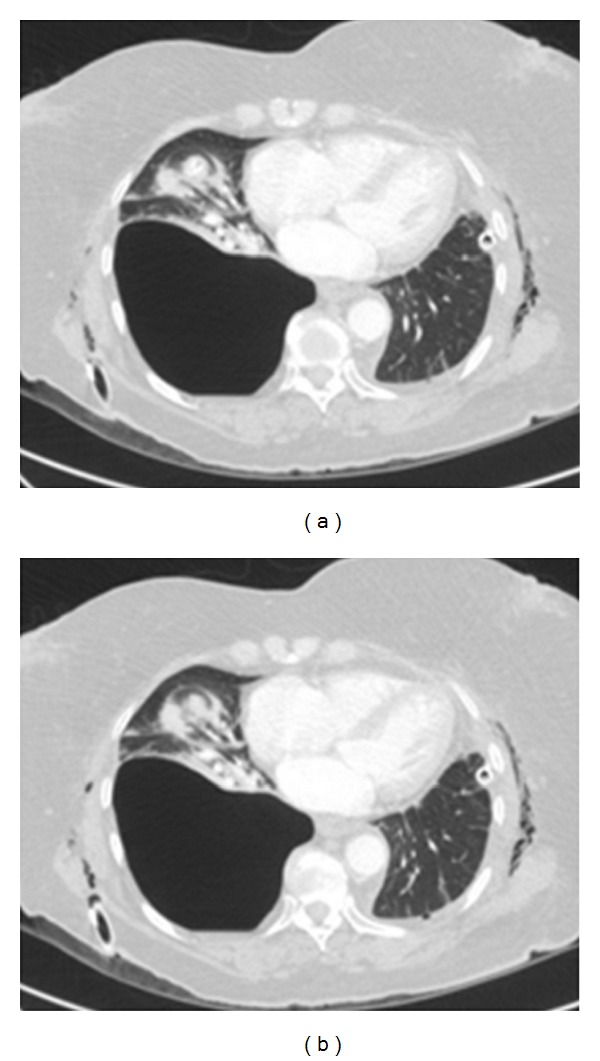
Axial CT scans on the same patient as in Figure 29 showing a large bulla in the right lower lobe associated with passive atelectasis in the right middle lobe. Note the surgical emphysema.

**Figure 19:**
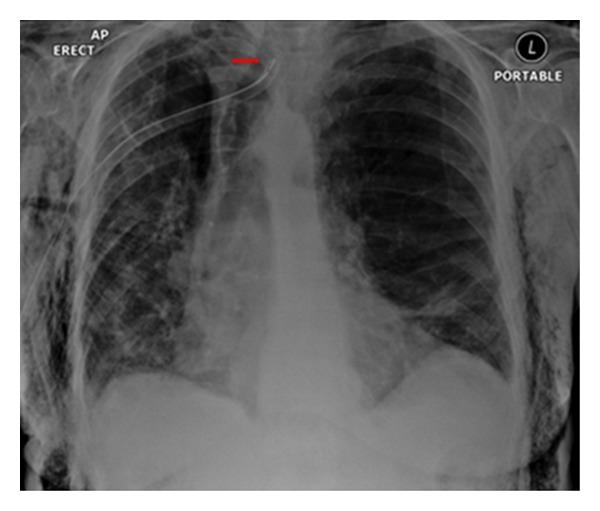
A CXR showing an inappropriately placed chest drain. Note the extensive surgical emphysema.

**Figure 20:**
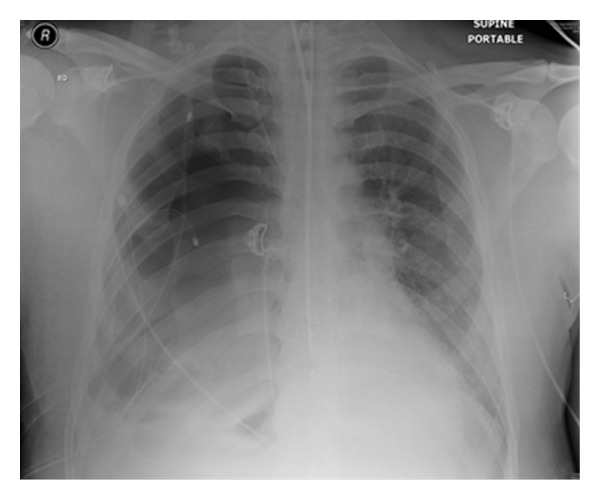
A CXR shows right-sided chest drain tension.

**Figure 21:**
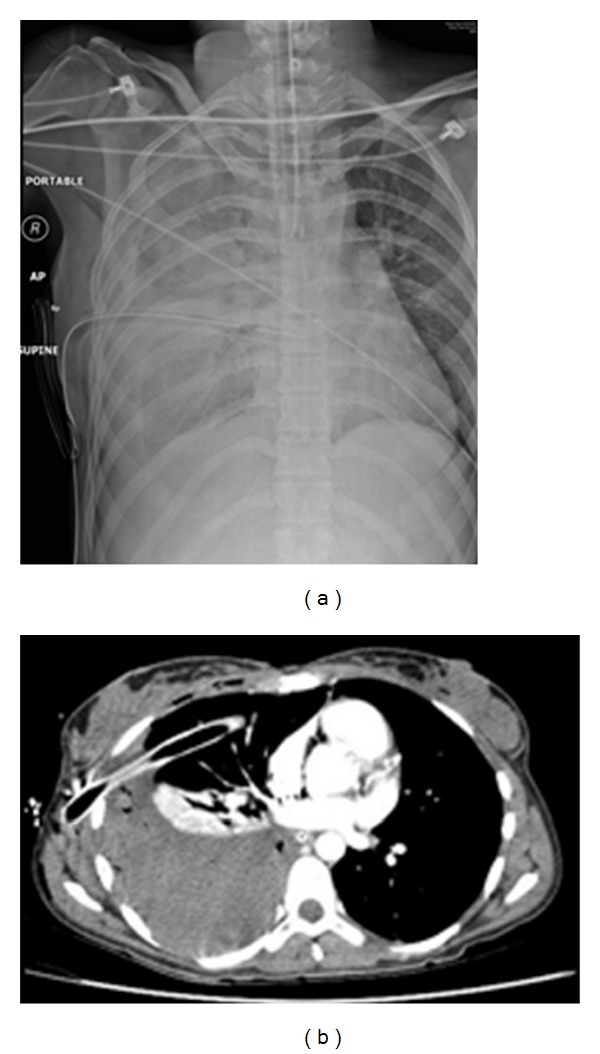
A CXR and axial CT show an inappropriately placed chest drain in an attempt to drain a right basal pleural effusion.

**Figure 22:**
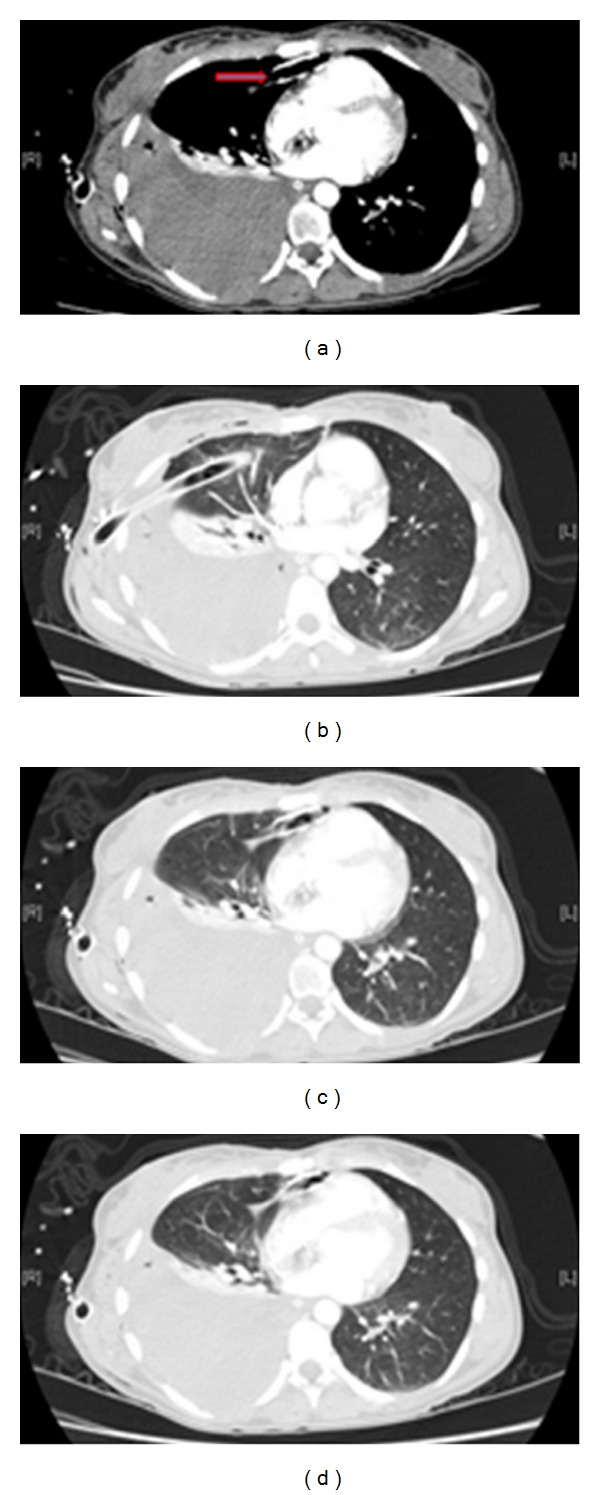
Axial CT scans on the same patient as in Figure 22 showing inappropriately placed chest drain in an attempt to drain a right basal pleural effusion.

**Figure 23:**
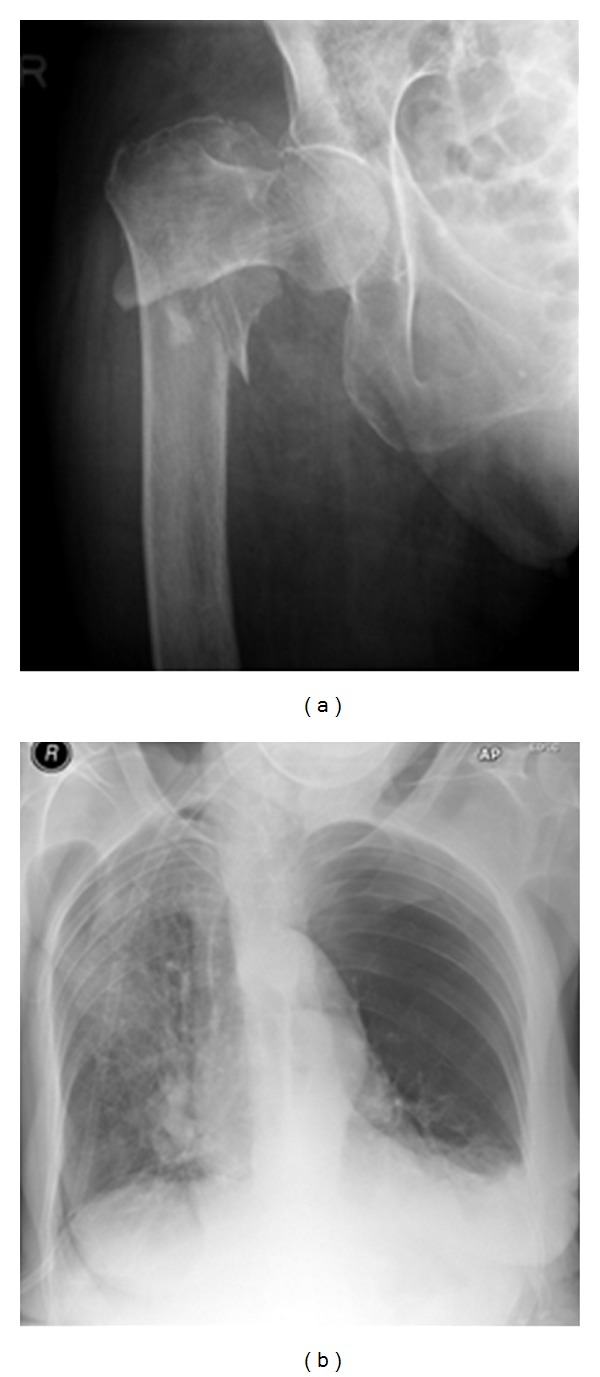
A hip and chest radiograph on an 87-year-old woman following a fall showing a fracture of the neck of the right hip. The CXR was interpreted as showing a left-sided pneumothorax, and thus, a chest drain was put in place (see Figures 25 and 26).

**Figure 24:**
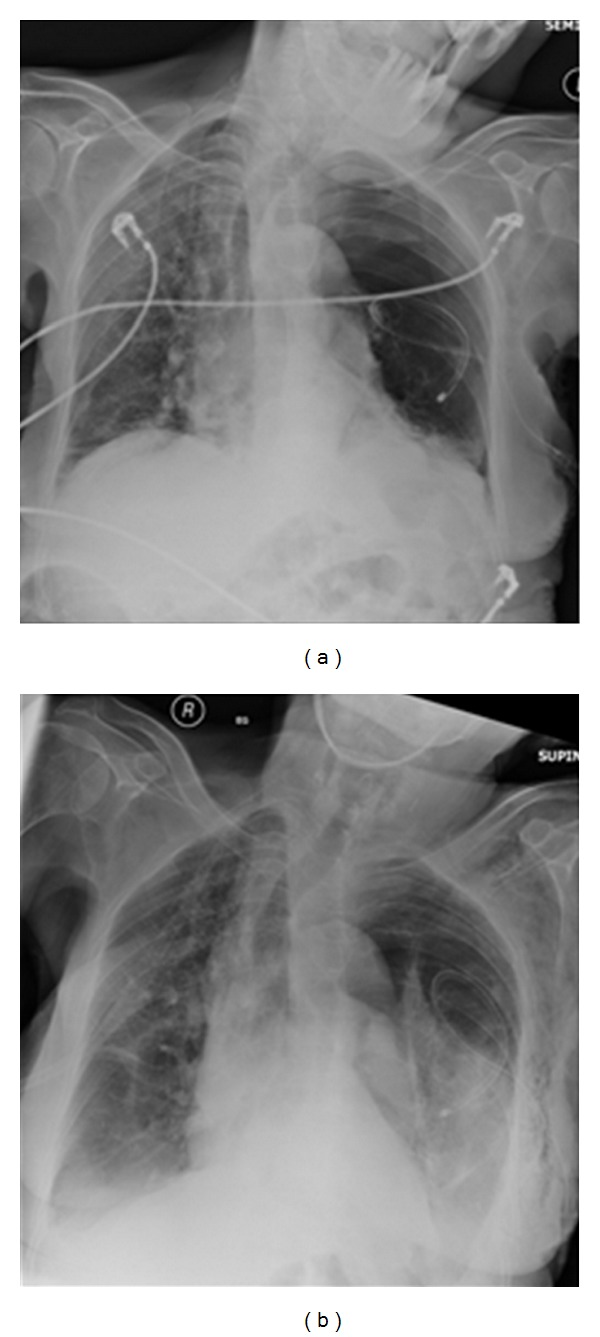
Two consecutive radiographs on the same patient as in Figure 24 showing a chest drain in situ at the left lung base. The patient's symptoms worsened following insertion of the chest drain. A repeat chest radiograph shows an accumulation of fluid at the left lung base.

**Figure 25:**
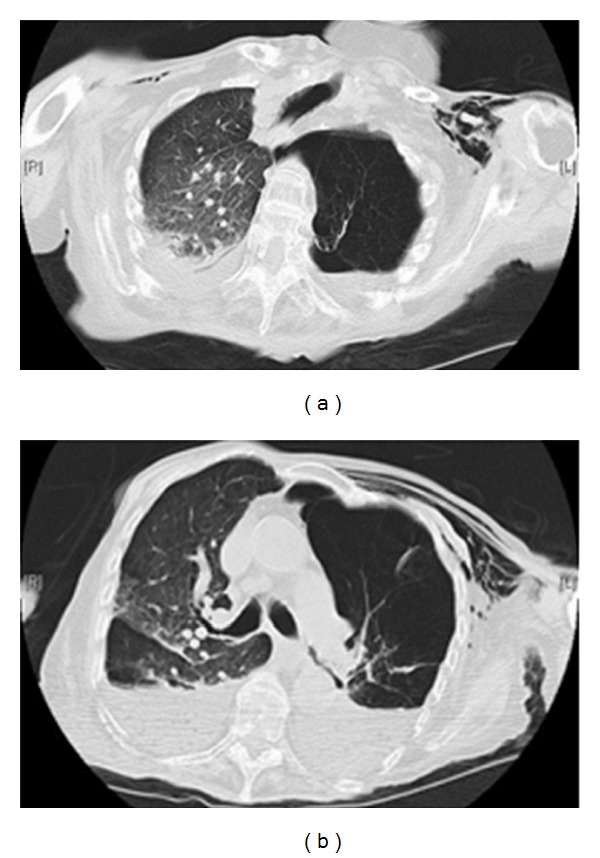
Axial CT scans on the patient in Figures 24 and 25 show that the chest drain was placed in bullous emphysema.

**Figure 26:**
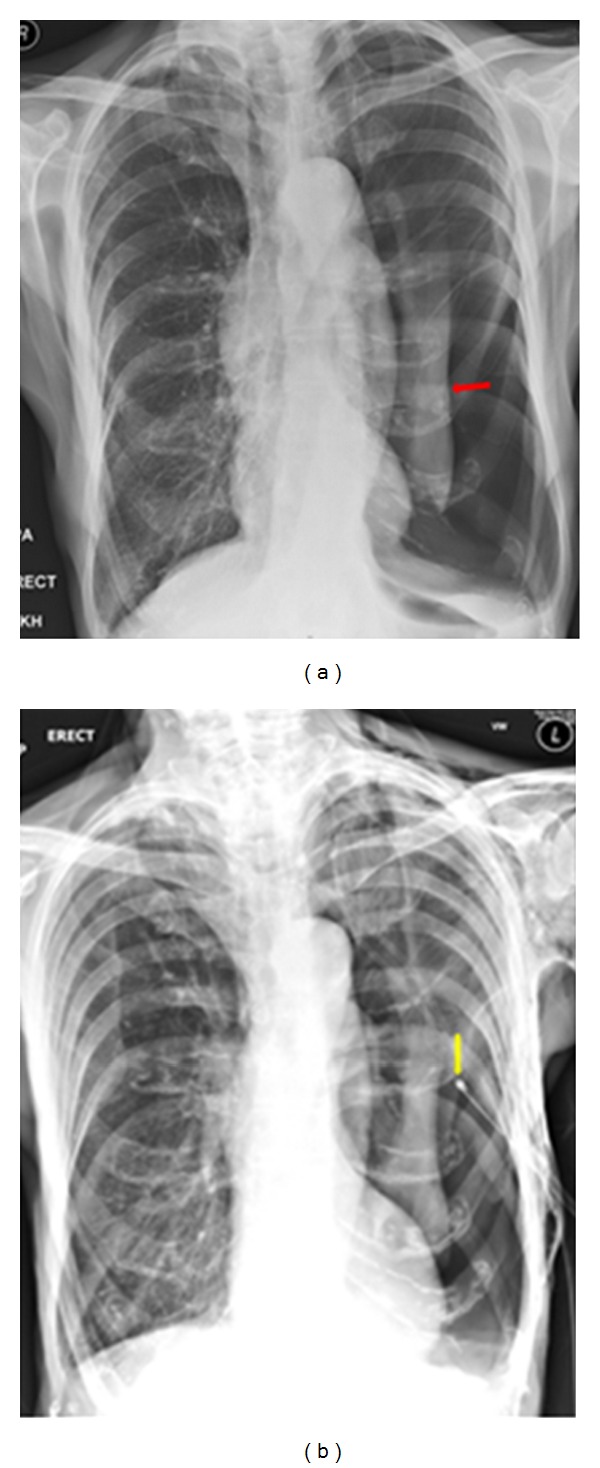
Reexpansion pulmonary edema (REPE) is a rare complication occurring after the insertion of a chest tube for pneumothorax or pleural effusion. REPE can appear on the ipsi- or contralateral side, can be bilateral and can even be asymptomatic. The case illustrated developed an ipsilateral REPE over a period of 6 hours following placement of a chest drain for a pneumothorax. The patient stabilized under continuous oxygen (12 L/min via a nonrebreather facemask) with his oxygen saturation steadily increasing. The patient required no further treatment. REPE is a serious complication associated with mortality of approximately 20%. Also, see Figure 27.

**Figure 27:**
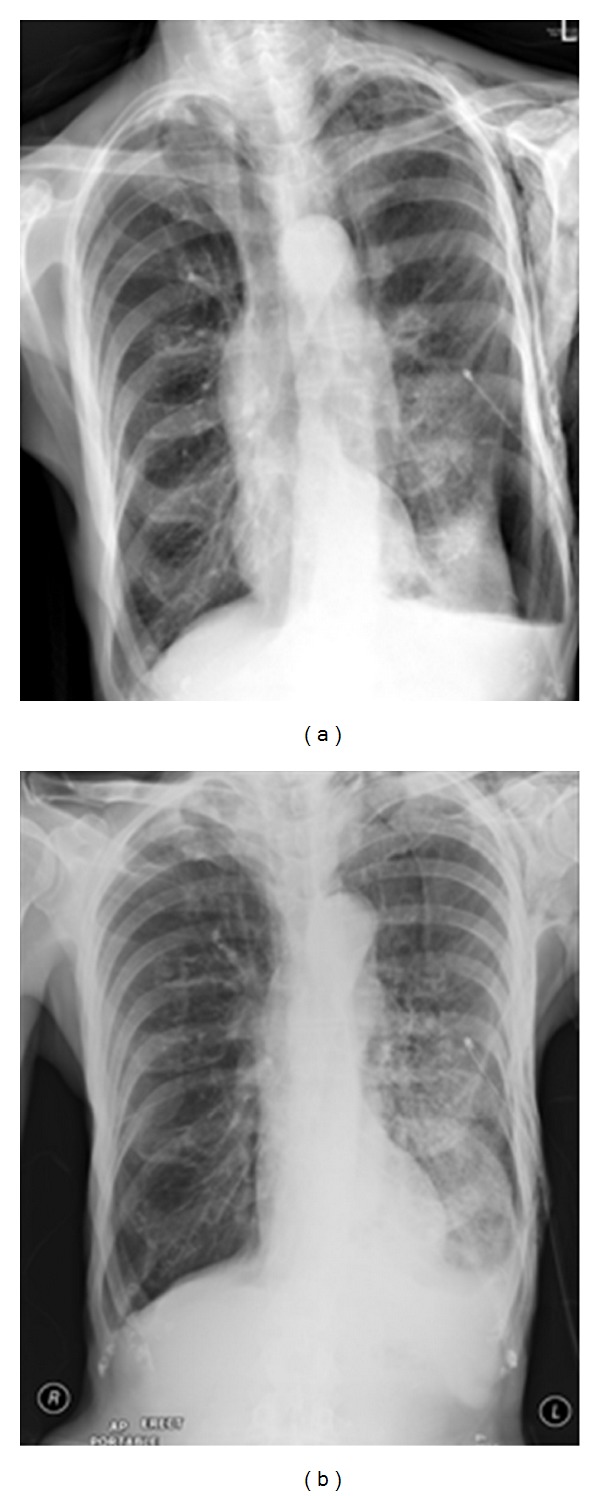
Reexpansion pulmonary edema (REPE) is a rare complication occurring after the insertion of a chest tube for pneumothorax or pleural effusion. REPE can appear on the ipsi- or contralateral side, can be bilateral and can even be asymptomatic. The case illustrated developed an ipsilateral REPE over a period of 6 hours following placement of a chest drain for a pneumothorax. The patient stabilized under continuous oxygen (12 L/min via a nonrebreather facemask) with his oxygen saturation steadily increasing. The patient required no further treatment. REPE is a serious complication associated with mortality of approximately 20%. Also, see Figure 27.

**Figure 28:**
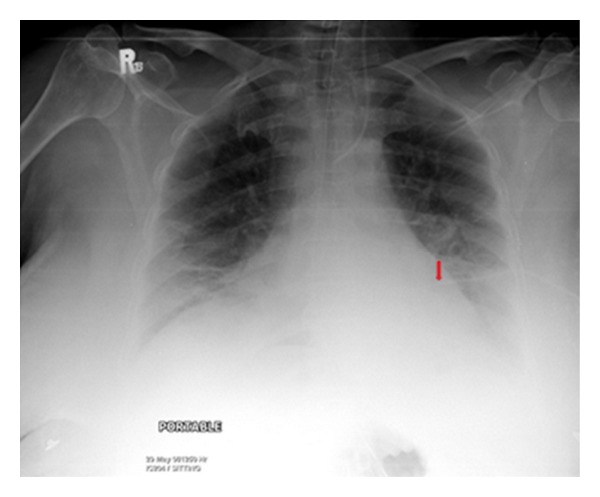
It shows a portable chest radiograph on an intensive care patient that developed pleural effusions. A chest drain was placed to drain the left-sided pleural effusion. Bright red blood was obtained from the tube. A CT scan (Figure 30) obtained immediately revealed the tip of the catheter had entered the left ventricle. The patient was immediately taken for cardiac surgery and the tube was removed without complications.

**Figure 29:**
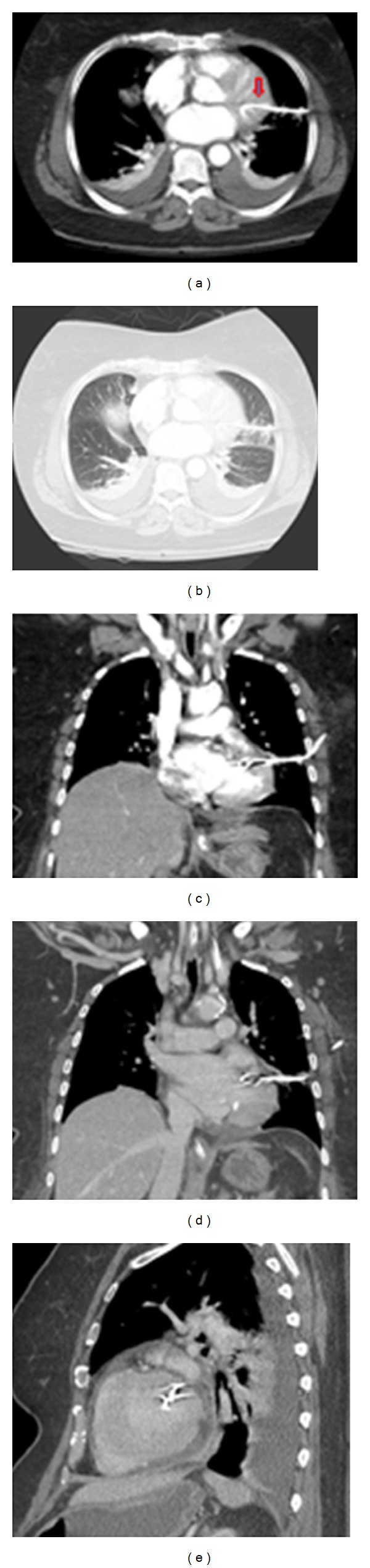
It shows a portable chest radiograph on an intensive care patient that developed pleural effusions. A chest drain was placed to drain the left-sided pleural effusion. Bright red blood was obtained from the tube. A CT scan (Figures 28 and 30) obtained immediately revealed the tip of the catheter had entered the left ventricle. The patient was immediately taken for cardiac surgery and the tube was removed without complications.

**Figure 30:**
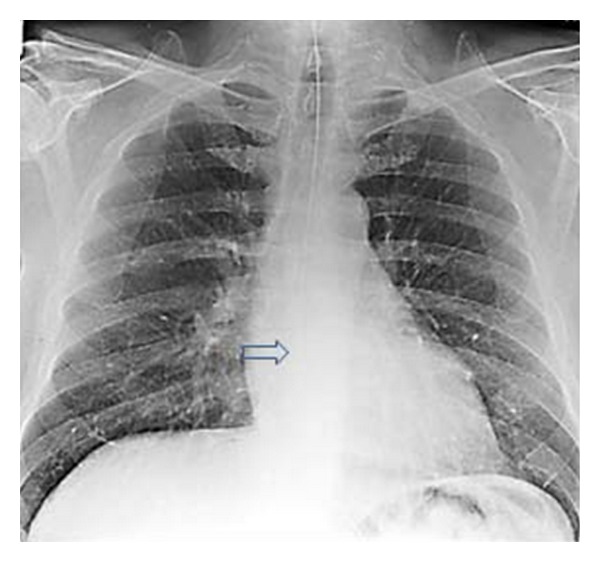
A CXR showing the tip of the NGT in the mid/lower esophagus. And it shows a portable chest radiograph on an intensive care patient that developed pleural effusions. A chest drain was placed to drain the left-sided pleural effusion. Bright red blood was obtained from the tube. A CT scan (Figure 28) obtained immediately revealed the tip of the catheter had entered the left ventricle. The patient was immediately taken for cardiac surgery and the tube removed without complications.

**Figure 31:**
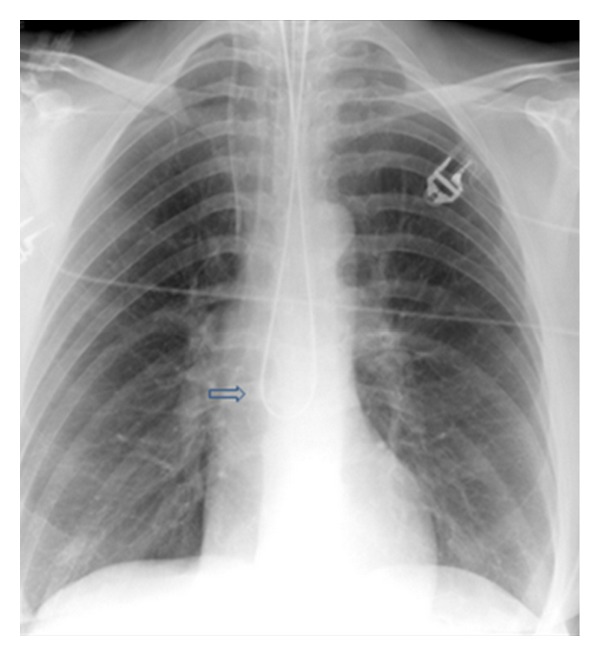
A CXR shows an NGT tube doubled up in a “hair pin” (arrow) fashion with reentry of its tip into the oropharynx.

**Figure 32:**
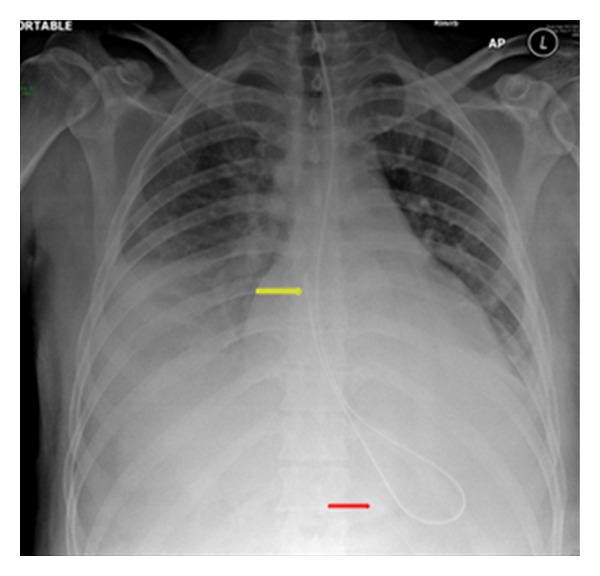
The NGT has formed a loop (red arrow) in the stomach and re-entered the mid/lower esophagus (yellow arrow). This complication may cause reflex in a supine patient and aspiration as in this patient.

**Figure 33:**
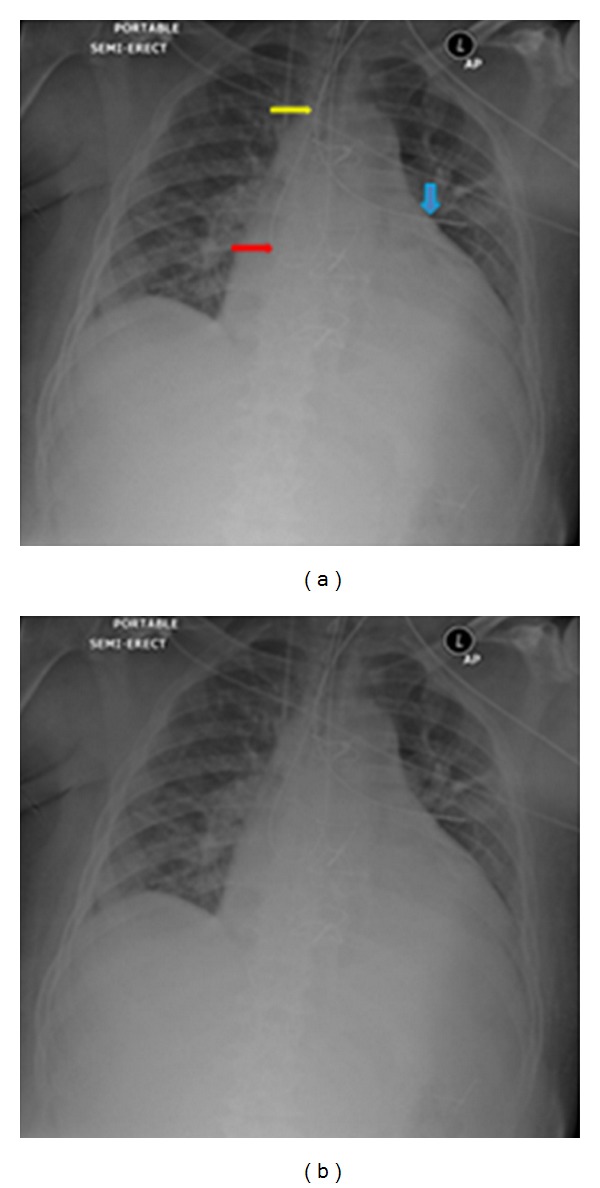
The NGT loops in the trachea (red arrow), then reenters the oropharynx, makes a further loop in the oropharynx, and returns to the trachea (yellow arrow), and finally the tip ends into the left, upper lobe bronchus probably the lingular bronchus (blue arrow).

**Figure 40:**
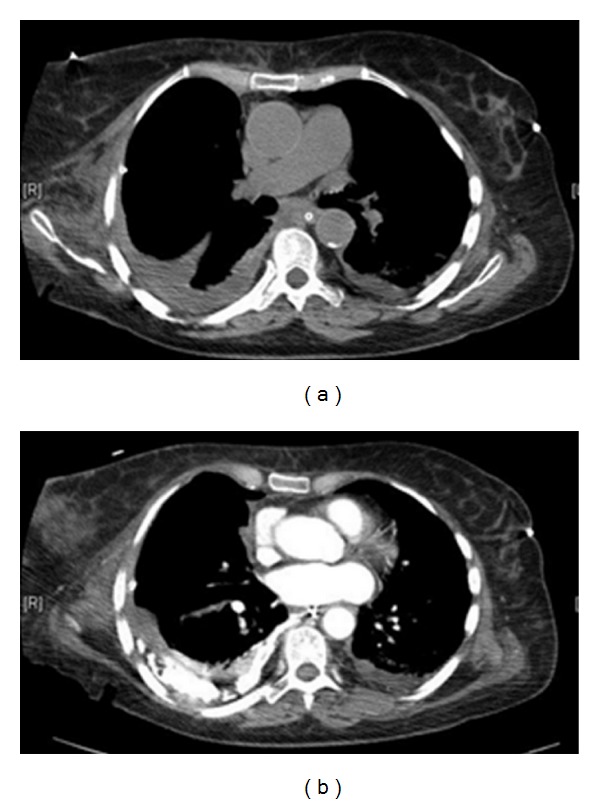
Axial CT scans of the same case as Figure 38 showing entry of water-soluble contrast into the right pleural space following esophageal perforation from an NGT.

**Figure 41:**
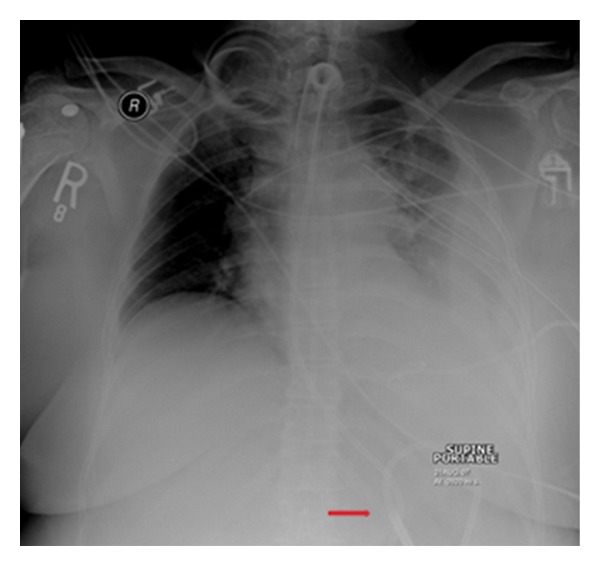
A CXR showing an NGT forming a loop within the fundus of the stomach.

**Table 1:** Central venous lines do's and donot's [28, 62].

(i) Do use to gain peripheral venous access, deliver substances not safely given via peripheral IV access, hemodialysis, plasmapheresis, measurement of cardiac filling pressures, placement of pulmonary artery catheter, placement of trans venous pacer and access for frequent blood sampling.	
(ii) Do not use if operator inexperienced, uncooperative patients and uncorrected coagulopathy.	
(iii) Do not use or use with caution in cellulitis/infected at anticipated insertion site, previous surgery/injury SVC, severe respiratory disease that cannot tolerate a pneumothorax (consider femoral route), when adequate peripheral access is available, vasculitis, congenital heart disease, presence of cardiac pacemaker and or other intracardiac devices.	
(iv) Do get informed consent for elective placement. In an emergency, do document the need in records.	
(v) Do make sure all materials are within reach before the commencing the procedure.	
(vi) Do use sterile precautions to reduce infective complications.	
(vii) Do use ultrasound guidance as it reduces the failure rate, especially for cannulation of the internal jugular vein.	
(viii) Do leave dilator in situ if you have entered an artery and call vascular surgeon.	
(ix) Do use large-bore catheters if rapid volume deliver is required.	
(x) Do remember that right internal placement with ultrasound guidance has a lower risk of pneumothorax than subclavian line placement.	
(xi) Do remember that central line placement in the femoral veins carries a higher risk of thrombotic and infectious complications.	
(xii) Do remember that there is a higher risk of air embolism in patients spontaneously breathing with large negative intrathoracic pressures, low CVP.	
(xiii) Do remember that arrhythmias are related to malpositioned catheter tip within right atrium or ventricle, and it resolves with pulling back of guidewire or catheter.	
(xiv) Do minimize thrombotic complications by ensuring that the catheter tip is located centrally within the distal third of the SVC or at the cavoatrial junction.	
(xv) Do prevent guidewire embolization. Keep your hand on the wire when possible and never loose site of the guidewire during the insertion process.	
(xvi) Do remember that incidence of arterial puncture is higher in pulseless patients, and remember veins are compressible.	
(xvii) Do obtain a chest X-ray following the procedure, even if unsuccessful line.	
(xviii) Do check the chest X-ray for line tip placement, pneumothorax, and hemothorax.	

**Table 2:** Dos and Don'ts of intercostal chest drain. BTS Guidelines [44, 50].

(i) Do use tension pneumothorax (PT) after initial needle relief, recurrent PT, in ventilated patients and large secondary spontaneous PT in patients over 50 years.	
(ii) Do use malignant pleural effusion, empyema, traumatic hemopneumothorax, and post-op pleural effusions.	
(iii) Do not use uncorrected coagulopathy and lung densely adherent to the chest wall throughout the hemithorax.	
(iv) Do not drain a postpneumonectomy space until consultation with a cardiothoracic surgeon.	
(v) Beware of lung bullous disease and do not confuse with PT and a lung collapse presenting as chest radiograph shows a unilateral “whiteout.”	
(vi) Do obtain informed consent and premedicate appropriately.	
(vii) Do aseptic technique and make sure all necessary equipment is at hand.	
(viii) Do insert the mid axillary line in the “safe triangle” with the patient in bed, slightly rotated, with the arm on the side of the lesion behind the patient's head. Alternatively, use upright sitting position with the patient leaning over a table with a pillow or in the lateral decubitus position.	
(ix) Do not insert drain without further image guidance if free air or fluid cannot be aspirated with a needle at the time of anesthesia.	
(x) Do use image guidance preferably ultrasound.	
(xi) A CXR must be available at the time of drain insertion except in the case of tension pneumothorax.	
(xii) 10–14 French (F) drains are generally used but larger bore catheters are preferred for a hemothorax.	
(xiii) Do avoid substantial force during insertion use a Seldinger technique or by blunt dissection through the chest wall and into the pleural space before catheter insertion.	
(xiv) Do insert a finger before inserting the intercostal catheter.	
(xv) Do not proceed if pulsatile bright red blood comes from the drain.	
(xvi) The position of the tip of the chest tube should ideally be aimed apically for a pneumothorax or basally for fluid.	
(xvii) Use +“Purse string” sutures to secure drains.	
(xviii) Never clamp a bubbling chest drain.	
(xix) Do a controlled drainage of large PEs.	
(xx) Avoid clamping CD in pneumothorax.	
(xxi) If a patient with a clamped CD develops breathlessness or subcutaneous emphysema, the drain must be immediately unclamped.	
(xxii) All chest tubes should be connected to a single flow drainage system, for example, under water seal bottle or flutter valve.	
(xxiii) Use of a flutter valve system allows earlier mobilization and the potential for earlier discharge of patients with chest drains.	

**Table 3:** Dos and Don'ts of NG Tube Placements [63].

(i) Do not intubate some patients with maxillofacial disorders, following maxillofacial surgery or trauma, esophageal tumors or surgery, laryngectomy, oropharyngeal tumors, skull fractures, unstable cervical spinal injuries (involving vertebrae 4 or above), and esophageal varices.	
(ii) Do explain the procedure to the patient.	
(iii) Do wear nonsterile gloves.	
(iv) Do wear a mask, eye protection, and a gown when dealing with patients prone to vomiting.	
(v) Be ready to apply suction when gaging/vomiting occurs.	
(vi) Do sit patients upright for optimal neck/stomach alignment if possible.	
(vii) Do examine the nostrils for obstruction; use the best side for intubation.	
(viii) Do measure tube from bridge of nose to earlobe, and to halfway between the inferior part of the sternum and the umbilicus.	
(ix) Do mark measured length with a marker.	
(x) Do lubricate 2–4 inches of the tube with Xylocaine (2%) jelly, squirt jelly in the nostril, and a spray of back of the throat with Xylocaine.	
(xi) Do partial prefreeze the NG tube to ease its passage.	
(xii) Do not rely on a cuffed endotracheal tube to prevent passage into the trachea.	
(xiii) Do pass the tube posteriorly via the nostril, past the pharynx into the esophagus and then the stomach and advance tube until the mark.	
(xiv) Do not advance tube against resistance.	
(xv) Do encourage the patient to swallow while advancing the tube.	
(xvi) Do facilitate swallowing with ice chips or water.	
(xvii) Do withdraw the tube immediately if patients experience respiratory distress, or if the tube coils in the mouth.	
(xviii) Do check position of the tube by syringe aspirating gastric contents.	
(xix) Do not inject air bolus.	
(xx) Do test the pH of the aspirated contents, which should below 6.	
(xxi) Do not rely on PH in patients on antacids, H2 antagonists, and proton pump inhibitors.	
(xxii) Do obtain a radiograph before delivering feeding/medication.	
(xxiii) X-ray confirmation is only valid at the time of the X-ray.	
(xxiv) Do secure tube with tape or similar holding device.	
(xxv) Do document the reason, the size, and type of tube used and the nature and amount of aspirate.	
(xxvi) Do heck manufacturer's instructions regarding length of time tube can be left in situ.	
